# Carnivore space use behaviors reveal variation in responses to human land modification

**DOI:** 10.1186/s40462-024-00493-7

**Published:** 2024-07-18

**Authors:** Nicole T. Gorman, Michael W. Eichholz, Daniel J. Skinner, Peter E. Schlichting, Guillaume Bastille-Rousseau

**Affiliations:** 1 Cooperative Wildlife Research Laboratory, Southern Illinois University, Carbondale, IL USA; 2 Department of Fish and Wildlife Conservation, Virginia Tech, Blacksburg, VA USA; 3 School of Biological Sciences, Southern Illinois University, Carbondale, IL USA; 4 Illinois Department of Natural Resources, Springfield, IL USA

**Keywords:** Animal movement, Spatial ecology, Carnivore, Bobcat, Coyote, Resource selection, Human land modification, Functional response

## Abstract

**Background:**

Spatial behavior, including home-ranging behaviors, habitat selection, and movement, can be extremely informative in estimating how animals respond to landscape heterogeneity. Responses in these spatial behaviors to features such as human land modification and resources can highlight a species’ spatial strategy to maximize fitness and minimize mortality. These strategies can vary on spatial, temporal, and individual scales, and the combination of behaviors on these scales can lead to very different strategies among species.

**Methods:**

Harnessing the variation present at these scales, we characterized how species may respond to stimuli in their environments ranging from broad- to fine-scale spatial responses to human modification in their environment. Using 15 bobcat-years and 31 coyote-years of GPS data from individuals inhabiting a landscape encompassing a range of human land modification, we evaluated the complexity of both species’ responses to human modification on the landscape through their home range size, habitat selection, and functional response behaviors, accounting for annual, seasonal, and diel variation.

**Results:**

Bobcats and coyotes used different strategies in response to human modification in their home ranges, with bobcats broadly expanding their home range with increases in human modification and displaying temporal consistency in functional response in habitat selection across both season and time of day. Meanwhile, coyotes did not expand their home ranges with increased human modification, but instead demonstrated fine-scale responses to human modification with habitat selection strategies that sometimes varied by time of day and season, paired with functional responses in selection behaviors.

**Conclusions:**

These differences in response to habitat, resources, and human modification between the two species highlighted the variation in spatial behaviors animals can use to exist in anthropogenic environments. Categorizing animal spatial behavior based on these spatiotemporal responses and individual variation can help in predicting how a species will respond to future change based on their current spatial behavior.

**Supplementary Information:**

The online version contains supplementary material available at 10.1186/s40462-024-00493-7.

## Background

Spatial behavior can be informative about how animals respond to the heterogeneity in their environment. These responses include broad-scale decisions about the size and location of the home range [10], as well as finer scale responses to heterogeneity in the environment through habitat selection decisions [34]. Animals often vary in their spatial behavior, which can be due in part to individual personality [35, 68] or behavioral plasticity [81, 83]. This variation can lead to the degree of selection of resources being dependent on the availability of resources in their environment, known as functional responses [25, 56], which can include responses to habitat [59], food resources [28, 82], or other stimuli. Functional responses in spatial behavior relative to anthropogenic features and activity have been documented in various species, specifically for habitat selection and proximity to humans in wolves (*Canis lupus*) [25], caribou (*Rangifer tarandus caribou*) [53], and moose (*Alces alces*) [7]. Habitat selection is particularly informative about a species' ecology because of the direct link between the spatial choices an individual makes and variability in the environment [33], and because it can have direct impact on reproduction and survival [52, 62]. Habitat selection and functional responses are especially important in the context of anthropogenic change, through which landscape composition is constantly undergoing modification.

Animals can display a wide range of responses to anthropogenic features. These responses can be broad-scale, such as consistently avoiding human activity or structures in their home range [40, 54], and which could lead to increased home range size for individuals inhabiting areas with greater levels of human land modification [63]. In contrast, species can also display fine-scale, nuanced responses to humans, such as only avoiding anthropogenic features within the home range and during a certain periods of the day [86], seasonally [32], or by using a combination of spatiotemporal responses [37]. These broad- and fine-scale behaviors do not only refer to the spatial scale (e.g., home range vs. landscape level) of the response, but also to the amount of spatial, temporal, and individual variation and complexity in their space use behaviors in response to a stimulus in their environment. Investigating individual and temporal variation in spatial behavior can elucidate broader patterns in behavior, linking spatial ecology and animal behavior [27], as well as help draw conclusions about population-level relationships with habitat [5]. Here, we propose that animals can vary in their behaviors on spatial, temporal, and individual axes at multiple scales in response to features on the landscape. These behaviors can be used to characterize animal responses to anthropogenic modification in their environment. Additionally, these responses can be particularly useful for wide-ranging species which use a variety of habitats with varying levels of human development.

Bobcats (*Lynx rufus*) and coyotes (*Canis latrans*) are two carnivores that fill the role of top predator in the absence of large predators throughout much of North America [38, 42, 76]. Bobcats are strictly carnivorous and are thought to prefer habitats with ample cover, such as forest when available [42, 44]. Coyotes are more generalist in both diet and habitat and are found in all habitats along a forested-to-agricultural gradient [42, 67], and are more likely than bobcats to exploit agricultural landscapes [44, 61]. Both bobcats and coyotes have the potential to maintain larger home ranges in the presence of fragmentation, but coyotes are more plastic and adaptable to anthropogenic change, exploiting small resource patches on a landscape scale, regardless of connection [1, 73, 87]. Although small carnivores are a group expected to adapt better than other taxa to human development [13, 84], carnivore species vary in their ability to coexist with humans based on flexibility in diet and suitable habitat, as well as plasticity in behaviors like boldness and neophilia, leading to a variety of responses to anthropogenic land modification [50, 69].

Here we studied how variation in anthropogenic land modification shapes spatial behaviors of two carnivore species and characterized the scale and complexity at which they respond to human modification. Specifically, we investigated how a gradient of human land modification impacted home range size and habitat selection of both species. We also evaluated how habitat selection behaviors varied temporally by season and time of day and how individual variation in these behaviors could be linked to variation to the intensity of human modification for an individual (functional response). Overall, given the behavior of both carnivores, we expected bobcat responses to be marked by stronger and more consistent avoidance of human modification and overall larger home range when exposed to human modification due to their tendency to be more avoidant of human impact than coyotes. Meanwhile, we expected coyote responses to be more fine-scale, with home range size less affected by human modification, but with space use showing more individual variation, temporally-dependent selection behaviors, and complexity in their functional responses to human modification.

## Methods

### Study area

Our study occurred at two sites in Illinois. The southern Illinois study site consists of Touch of Nature Environmental Center (37.62762, − 89.15827) and Giant City State Park (37.60195, − 89.18925), making up a combined 28.6 km^2^ of land managed by Southern Illinois University and the state of Illinois. The landscape at the southern study site is largely forested (Jackson and Union Counties, Illinois, consist of 40% agriculture and 41% forest), with an average annual temperature of 14.1 °C and an average annual precipitation of 118 cm [58]. The central Illinois study site consists of properties managed by the state of Illinois and U.S. Army Corps of Engineers surrounding Lake Shelbyville (39.51856, − 88.70658). The landscape is dominated by row crop corn and soybean agriculture, with some lakeshore, small towns, and remnant forested patches (Moultrie and Shelby Counties, Illinois, consist of 79% agriculture and 12% forest). The central Illinois study site has an average annual temperature of 12.2 °C and average annual precipitation of 120 cm [58].

### Capture and Handling

Bobcats and coyotes in both study sites were captured using cage traps (Tomahawk Live Trap, Hazelhurst, Wisconsin, Model 209.5, and homemade traps with similar dimensions, [6]) and rubber-padded foothold traps (Minnesota Trapline Products, Pennock, Minnesota, MB-650-RJ, [80]) during four winter capture seasons from January 2018 through March 2021. Bobcats were chemically immobilized with ketamine and xylazine and recovered inside a cage trap before release (ZooPharm, [6]). Coyotes were chemically immobilized with BAM™ (butorphanol tartrate, azaperone, and medetomidine hydrochloride) and the BAM™ was reversed post-handling with naltrexone and atipamezole before release (ZooPharm, [11]). All captured animals were fitted with LiteTrack Iridium 250 GPS collars (250 g, Lotek Wireless, Newmarket, Ontario, Canada) equipped with a release mechanism to drop off after one year. Thirty-two collars recorded GPS locations once every 1.5 h and 14 collars had a different schedule due to the GPS fix rate requirements of various project goals (1, 2, 3, or 4 h).

### Spatial covariates

Several spatial covariates were used in seasonal delineation and resource selection analyses. Landcover covariates were sourced from a 30 m resolution National Land Cover Database classification [88] and reclassified to combine them into six landcover categories (water, exurban [denotes development], grassland and scrub, forest, agriculture, and wetland) for seasonal delineation (see below for seasonal delineation details) and four landcover categories (exurban, forest, agriculture, and other) for resource selection analyses. Grassland, scrub, and wetland were rare and so were combined with the other wetland category to avoid including resources not used by some individuals. We also included a separate human land modification covariate using a global layer which quantifies the degree of human land modification on a continuous scale, using 13 anthropogenic global stressors under the categories of human settlement, agriculture, transportation, mining and energy production, and electrical infrastructure at a 1 km resolution (see [36] for full details on this layer). We reprocessed a layer of Illinois streams and shorelines [29] to create a Euclidean distance to water covariate at 30 m resolution and took the natural logarithm of the Euclidean distances to account for decreasing impact of a water source with increasing distance from it [41]. Similarly, we reprocessed a layer of Illinois paved roads [30] to create a natural logarithm of the Euclidean distance to road covariate at 30 m resolution.

### Home range size

To estimate the annual home ranges of bobcats and coyotes, we used autocorrelated kernel density estimation (AKDE), as developed by Fleming et al. [14]. We used the package ‘ctmm’ in Program R [12] to estimate home ranges. Home range sizes were calculated using the Ornstein–Uhlenbeck with foraging (OUF) model using a 0.95 quantile. Data from 11 individuals did not meet the requirements for AKDE and were excluded from home range size analyses, commonly due to instances of dispersal or short duration of tracking.

After log-transforming the home range sizes, we used a two-sample t-test to identify between-species differences in annual home range size [39, 41]. We performed similar tests between bobcat and coyote study sites and sexes, but due to uneven sample sizes between sex and study site within species, information on home range size comparisons by demographics can be found in Supporting Information. We used univariate linear models to evaluate how the degree of human modification, described above [36], in each home range impacted home range size in both species. We calculated the degree of human modification in each home range by averaging the human modification values within each home range and converting it from a 0 to 1 scale to a percentage. Intercept-only, linear, and quadratic regressions were performed and compared using the Akaike Information Criterion with correction for small sample size (AIC_C_) to determine the top regression model [9].

### Temporal period delineation

We used a clustering algorithm to define seasons ecologically in Program R [2]. To define bobcat and coyote seasons, the movement speed and turning angle between successive locations were calculated. Using a moving window of time, we calculated the mean speed and tortuosity, as well as the proportion of the locations in water, exurban, grassland and scrub, forest, agriculture, and wetland landcover areas within the moving window [88]. The DD-weighted gap method [92] was used to determine the optimal number of clusters (seasons). We then used K-means clustering analysis [24, 47] to identify clusters of similar space use behaviors to define seasons, adjusting bootstrap thresholds and windows of seasonal length to ensure continuous seasons of adequate length (longer than two weeks).

Day, night, and crepuscular diel periods were also delineated (diel period details in Supporting Information). Equinox and solstice dates [57] were used to divide the year into four periods, and the average sunrise and sunset time for each period was calculated [49] to account for changes in day length between the four periods [85]. Day was delineated as two hours after sunrise to one hour before sunset, night as two hours after sunset to one hour before sunrise, and crepuscular as the two lengths of time one hour before to two hours after sunrise and sunset [16, 31, 85]. The four species-specific seasons and three diel periods (day, night, crepuscular) were combined to create twelve bobcat temporal periods and twelve coyote temporal periods.

### Resource selection functions

To determine individual-level habitat selection, we used a logistic regression to estimate resource selection functions (RSFs) for each individual within the annual home ranges [5, 48]. The individuals that were previously excluded from home range size analysis were included in the RSFs, using kernel density estimation (KDE) annual home range estimations (instead of AKDE) [91]. Twelve thousand random locations were generated within each of these home ranges. Each random location was also assigned a random date and time sampled from the dates and times of the used points [3], and the previously described temporal periods were applied to each used and random point so that each point was categorized based on its season and diel period. Landcover categories, human modification, distance to water, and distance to road covariates were extracted for each used and random point. Landcover categories included the dummy variables of forest (reference category), agricultural, exurban, and other. The continuous variables of human modification, distance to water, and distance to road covariates were scaled (divided by one standard deviation) so they could be easily compared [77].

Bobcat and coyote RSFs were estimated using the package ‘IndRSA’ in Program R [5]. ‘IndRSA’ estimates an individual-level RSF for each individual and a population average in a second step [55]. Models were estimated for each permutation of species and temporal period. K-fold cross-validations were performed for each output (k = 5), and individuals with a k-fold value less than 0.2 were excluded from the results.

### Impacts of human modification on carnivore behavior

We estimated how human modification directionally affects bobcat and coyote habitat selection behavior in the form of functional responses in habitat selection [25, 53]. We used univariate regressions with the individual RSF coefficients for five covariates (agriculture, exurban, and other landcover; distance to water; and distance to road) as the response variables and the degree of human modification in each home range as the explanatory variables. These regressions were separated based on temporal period to discern temporal effects on the functional responses. In addition, regressions of the means of coefficients for each covariate during all temporal periods for each degree of human modification in home range (the mean selection of each covariate for each individual) were performed to find if a broad functional response was present regardless of temporal period. Weighted regressions were used to account for uncertainty associated with the RSF coefficients [4]. Intercept-only, linear, and quadratic regressions were compared using AIC_C_ to determine the top regression model [9]. The “other” landcover category lacked biological meaning and so was not included in the results but can be found in the Supporting Information.

## Results

Fifteen bobcat-years (central Illinois female *n* = 1, central Illinois male *n* = 3, southern Illinois female *n* = 6, southern Illinois male *n* = 5) and 31 coyote-years (central Illinois female *n* = 6, central Illinois male *n* = 17, southern Illinois female *n* = 5, southern Illinois male *n* = 3) of location data were collected. An average of 1,397 GPS locations were obtained from each bobcat (range 293–2,695) and an average of 1,736 locations were obtained from each coyote (range 213–3,596), dependent on the lifespan of the animal and the battery life of the collar (maximum one year). Details regarding data demographics, GPS fixes, and landcover category use for each animal can be found in the Supporting Information, Appendix 1. Fourteen bobcat-years and 28 coyote-years of GPS data where the individuals were tracked for over two months were used to calculate four bobcat and four coyote seasons. Bobcats had short, distinct seasons in fall, early winter, and late winter, but had one long season during spring and summer (season details in Supporting Information, Appendix 2). Coyotes had short seasons during early and late winter and two longer spring and summer/fall seasons.

### Home range size

**Fig. 1:**
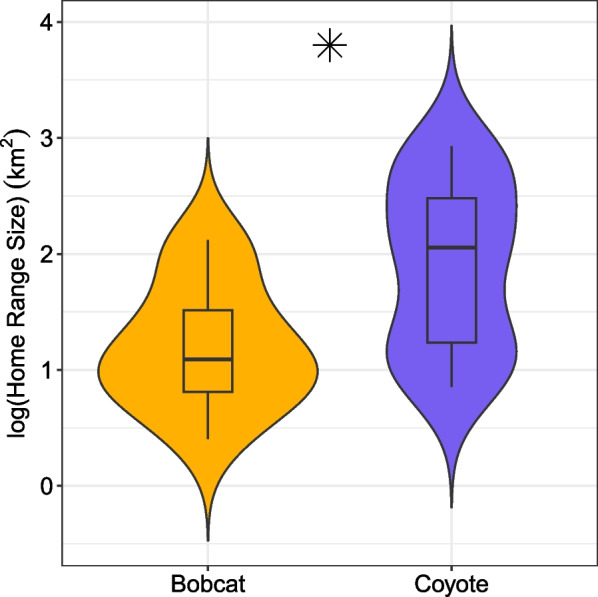
Population-level bobcat (orange) and coyote (purple) home range size estimations using AKDE. Asterisk indicates significant difference in home range sizes (α = 0.05)

Thirty-five annual home ranges were estimated using AKDE (Fig. 1). Bobcat mean home range size was 32.3 km^2^ (central Illinois male *n* = 2, southern Illinois female *n* = 6, southern Illinois male *n* = 3, range 2.5–132.0 km^2^) and coyote mean home range size was 221.4 km^2^ (central Illinois female *n* = 5, central Illinois male *n* = 13, southern Illinois female *n* = 5, southern Illinois male *n* = 1, range 7.1–849.0 km^2^) (details on home range size uncertainty in Supporting Information, Appendix 3). Bobcat home ranges were significantly smaller than those of coyotes (bobcat μ = 32.3 km^2^, coyote μ = 221.4 km^2^, t =  − 2.801, DF = 33, p = 0.009).

**Fig. 2:**
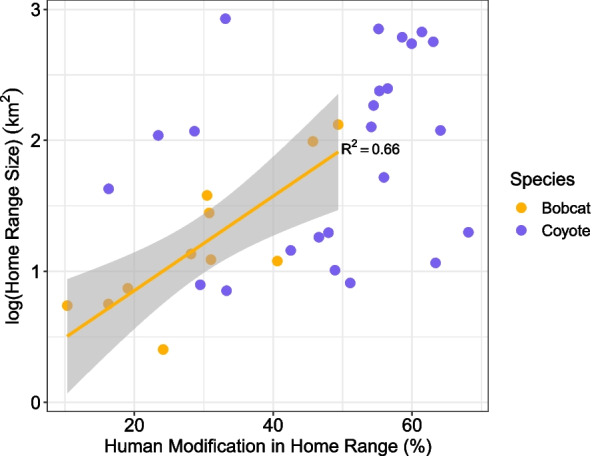
Individual bobcat and coyote home range size estimations paired with the degree of human modification within the home ranges. Bobcat home range size had a linear relationship with human modification (trendline and confidence interval shadow shown), while coyote home range size had no relationship with human modification (with no trendline to display)

The home range size of individual bobcats had a positive linear relationship with the degree of human modification within their home ranges (R^2^ = 0.66), with increased human modification being correlated with larger home ranges (Fig. 2). The intercept-only model was the top model for coyotes, indicating we found no relationship between the degree of human modification within home ranges and home range size (AIC_C_ table in Supporting Information, Appendix 4).

### Habitat selection

**Fig. 3:**
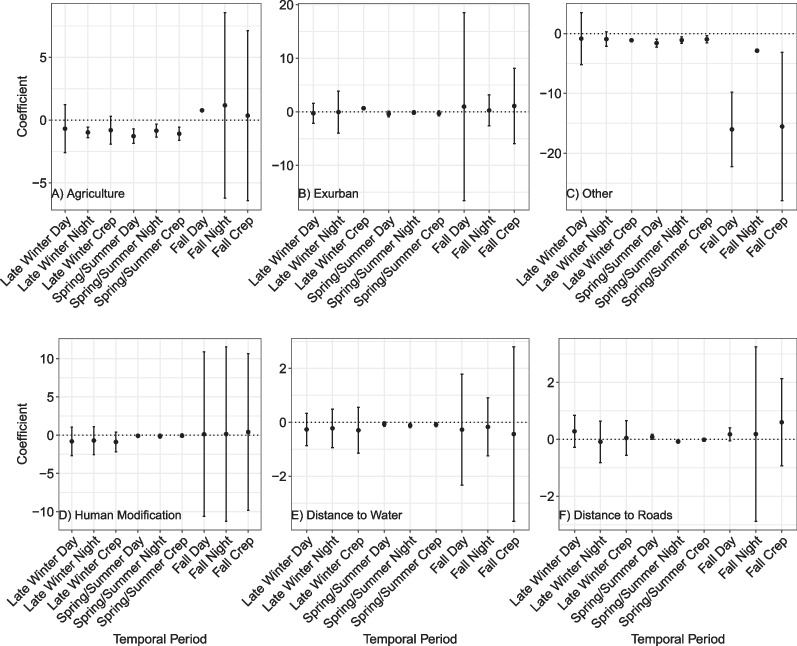
Population-level bobcat RSF coefficients of agricultural landcover, exurban landcover, other landcover, human modification, distance to water, and distance to road covariates in reference to forested landcover with 95% confidence interval bars. The results are divided by bobcat seasons (late winter, spring/summer, fall) and diel period (day, night, crepuscular) for coefficients representing nine temporal periods

No individual bobcats were tracked during each diel period for the entire duration of the early winter season, so that season was excluded from the bobcat RSF results. Bobcats avoided agriculture during the spring/summer season and late winter night, weakly avoided (confidence intervals overlapping 0) it during late winter day and crepuscular periods, weakly selected it during fall crepuscular and night periods, and selected it during fall day (*n* = 15, k-fold mean = 0.62, range 0.28–0.92) (Fig. 3). Bobcats generally did not respond to exurban habitat or human modification. Bobcats tended to select areas farther from roads and closer to water in periods when they had any response. The "other" landcover category lacks biological meaning, so from here onward we report the coefficients but will not interpret the meaning.

**Fig. 4:**
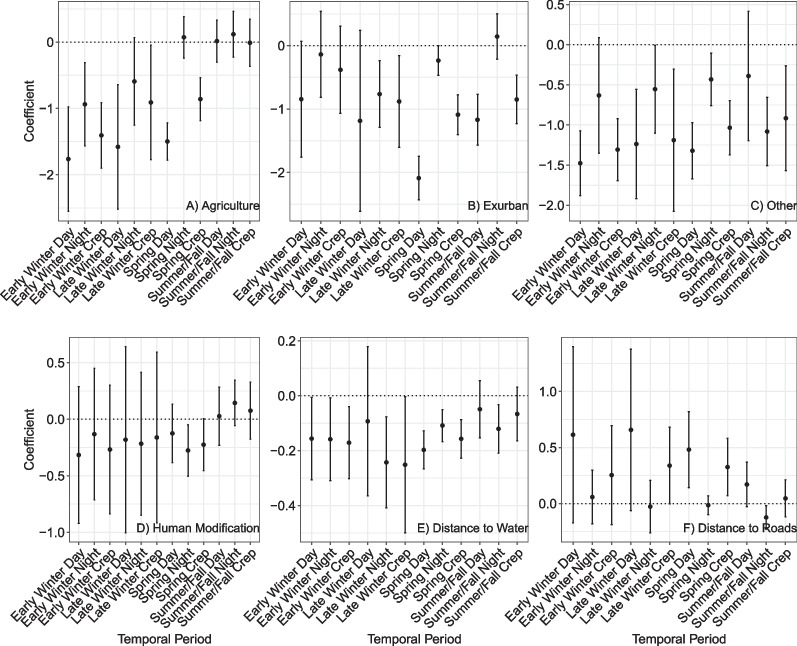
Population-level coyote RSF coefficients of agricultural landcover, exurban landcover, other landcover, human modification, distance to water, and distance to road covariates in reference to forested landcover with 95% confidence interval bars. The results are divided by coyote seasons (early winter, late winter, spring, summer/fall) and diel period (day, night, crepuscular) for coefficients representing twelve temporal periods

Coyotes generally avoided agriculture regardless of season, but the strength of avoidance varied by temporal period; avoidance decreased at night regardless of season (sometimes resulting in positive selection) (*n* = 31, k-fold mean = 0.67, range 0.20–0.96) (Fig. 4). Coyotes generally avoided exurban habitat, with weak avoidance at night and stronger avoidance during the day and crepuscular periods, regardless of season. Coyote avoidance of most landcover categories during most temporal periods indicated they mainly preferred forest over alternative habitat types. Coyotes generally did not select for or avoid human modification. They generally selected areas closer to water and weakly selected areas farther from roads during most temporal periods.

### Impacts of human land modification on carnivore behavior

**Fig. 5:**
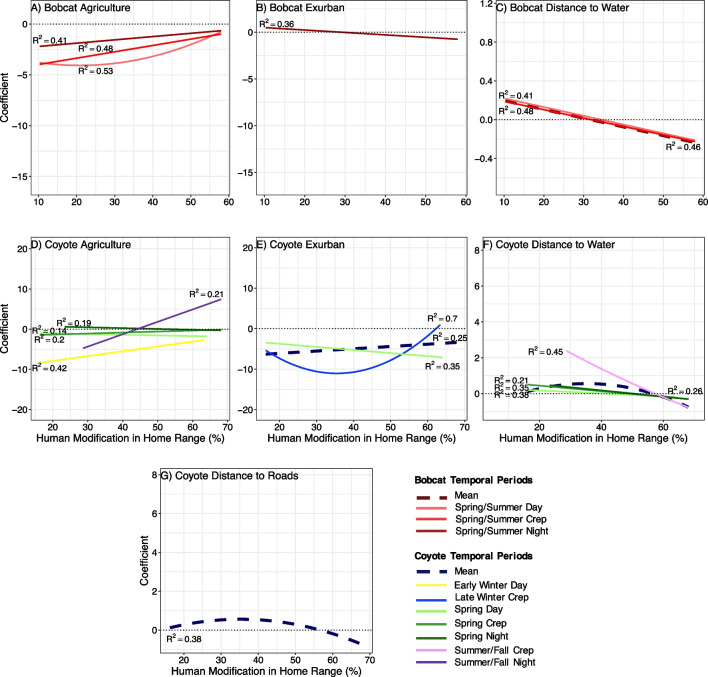
Top regression model trendlines for bobcat (**A**–**C**) and coyote (**D**–**G**) functional responses. Individual-level RSF coefficients for agricultural landcover, exurban landcover, distance to water, and distance to roads were regressed against the response to the degree of human modification present in each individual’s home range. Trendlines are displayed when the relationship was linear or quadratic and are not displayed if the relationship was intercept-only based on AIC_C_ model selection (see Supporting Information, Appendix 6). Trendlines are displayed for specific temporal periods (solid) or mean of all temporal periods (bolded and dashed). R^2^ values are displayed for each regression

We investigated whether there were functional responses between carnivore habitat selection and the degree of human modification in their home range (mean selection coefficients regardless of temporal period, hereafter referred to as “mean”), in addition to separating out selection by temporal period to gain insight about whether there was a temporal aspect to functional responses (Fig. 5). Bobcats exposed to higher degrees of human modification in their home ranges selected for more agriculture. There was a quadratic relationship in the spring/summer day period and a positive linear relationship in the spring/summer crepuscular and night temporal periods. Exurban selection and human modification exhibited a negative linear relationship during the spring/summer night, indicating that bobcats avoided exurban habitat with increased human modification during that period. Bobcats also exhibited negative linear relationships of human modification and distance to water in the mean and the spring/summer day and crepuscular periods, indicating selection to areas near water with increased human modification. Distance to road regressions yielded intercept-only top models for all temporal periods and means for bobcats, indicating no relationship between intensity of human modification and distance to road (AIC_C_ table in Supporting Information, Appendix 5).

Coyote selection for agriculture varied in response to the degree of human modification. Agriculture selection had a positive linear relationship in the early winter day, spring crepuscular, and summer/fall night periods and a negative linear relationship in the spring day and night periods with increasing human modification, indicating coyote functional responses may be dependent on season and/or time of day (Fig. 5). There was a positive linear mean relationship between coyote selection for exurban habitat and human modification, a negative linear trend in the spring day period, and a quadratic relationship in the late winter crepuscular period. Coyote selection had a mean quadratic relationship between human modification and distance to water, as well as slightly negative linear trends in spring day, crepuscular, and night periods. There was a strong negative linear trend in the summer/fall crepuscular period, indicating that, in general, coyotes selected areas farther from water as human modification increased. Coyotes displayed a quadratic relationship between distance to roads and human modification only in the mean, but not in any specific temporal periods.

## Discussion

Using a gradient of human modification within carnivore home ranges in two study sites, we aimed to better understand how landcover types and intensity of human modification affect carnivore behaviors both spatially and temporally. As hypothesized, we found notable differences between bobcats and coyotes in the degree of complexity in their responses to human modification. While differences in wildlife species’ response to human activity has been studied before (e.g., [17]), our work characterized specific responses to anthropogenic disturbances based on several behaviors across spatial and temporal contexts. Bobcat responses included larger home ranges with increased human modification within them in addition to weak habitat selection responses to agriculture, exurban areas, and human modification. Bobcats also displayed functional responses in their habitat selection choices that were relatively temporally consistent. In contrast, coyote home range size did not increase with human modification, but instead they displayed complex responses including stronger avoidance of agriculture, exurban areas, and human modification than bobcats, indicating more fine-scale avoidance behaviors within the home range. Coyote habitat selection functional responses were more nuanced, temporally-dependent, and sometimes changed direction depending on the amount of human modification (quadradic relationships). Our work provides evidence that species inhabiting the same landscape, and even filling a similar trophic role as top predators in this system, can vary widely in the degree of scale and complexity of their behavioral response to their environment. Specifically, our work shows the importance of investigating spatial and temporal variation in habitat selection and functional responses to better understand the complexity in how extrinsic factors shape wildlife behavior.

### Carnivore spatial behavior and response to human modification

Overall, higher degrees of human modification within home range were correlated with larger home range size in bobcats. Bobcats tend to use larger home ranges in more fragmented and developed landscapes [73, 87] and lynx (*Lynx lynx*) have been found to expand their home ranges to increase hunting efforts in areas with declining prey abundance [78]. Therefore, the fragmentated, patchy landscape and increased human modification in the central Illinois site could be leading to low-quality resources for bobcats, causing them to expand their home ranges to maintain access to necessary resources [61, 87]. Coyotes had larger home ranges than bobcats, but a large amount of variation was present within the coyote population [20, 23]. In addition, there was no correlation between human modification within their home ranges and home range size in coyotes. This lack of response is likely due to coyotes adjusting to human modification in their home ranges in other ways, such as spatial choices within their home ranges [19] or temporal adaptations to human activity [18, 79]. The great potential for behavioral plasticity in coyotes [8] could lead to the high levels of individual variation in home range size and adjustments within the home range that we observed.

Coyotes displayed a higher degree of temporal adjustment in their habitat selection than bobcats; their selection responses were more varied depending on the temporal period, both by time of day and by season, than bobcats. We observed bobcat tolerance of human modification and exurban habitat regardless of temporal period, which was unexpected based on previous studies (e.g., [70, 73]). Bobcats could be diluting the human density within their home ranges by expanding their home ranges in response to human modification, becoming less negatively affected by human modification and exurban areas overall. This dilution could be possible as long as human modification is below an animal’s threshold for acceptable modification within their home range [60, 64].

Bobcats and coyotes both adjusted their responses to agriculture, exurban habitat, and water depending on the degree of human modification around them. Bobcat functional responses to human modification in their home ranges were straightforward, selecting more agriculture, less exurban habitat, and areas closer to water as human modification increased. This means that human modification does impact bobcat behavior, causing them to adjust their use of habitat accordingly, which was expected based on other work (e.g., [15]). The directionality of these trends (positive or negative trend in selection with increasing human modification) was consistent when they were present regardless of the temporal period, although the strength of the trend sometimes varied by temporal period. Coyotes had a more varied response to human modification and mean annual responses did not always reflect the same trends present in specific temporal periods, although often trends within the same season were similar. These differences in functional response trends based on temporal period could be due to factors such as differences in prey availability depending on season or time of day in combination with human modification or variation in the availability of and need for cover during different seasons and times of day (e.g., selection for agriculture may increase during summer/fall when crops provide cover to avoid human activity in the presence of high human modification). In addition, regressions were sometimes quadratic and changed direction after a threshold of human modification, indicating coyotes may have more complex functional responses that completely changes response patterns when human modification becomes extremely prevalent in their home range (e.g., > 50% modified). Overall, coyote functional responses to human modification were more nuanced and temporally-dependent than those of bobcats.

### Variation of response to anthropogenic change

Taken altogether, these results support the idea that bobcats and coyotes respond differently to human modification in the scale and complexity of their space use behaviors, including home range size, habitat selection, and functional responses. Bobcats exhibited a broad-scale response to human modification. When faced with human modification, bobcats expanded their home ranges and functionally responded in their selection in a predictable manner with little temporal variation and complexity in their habitat selection overall. These results corroborate previous work that shows that bobcats avoid humans (e.g., [71]) and rely on corridors across a development gradient [51, 66]. In contrast, human modification did not affect coyote home range size, but it did cause coyotes to have more temporally-dependent habitat selection behaviors and varied and complex functional responses in their habitat selection, which often changed temporally in intensity or direction. Compared to bobcats, coyotes were able to fine-tune their spatial behavior by avoiding undesirable features on the landscape (e.g., exurban areas) on a finer scale within their home range instead of expanding their range. While it might be unanticipated that a species adapted to coexistence with humans would avoid human modification, this avoidance of human-associated areas [22] is a part of their adjustment strategy. Coyote temporal adjustments have been documented, including changing habitat preferences on a daily scale to avoid risk [65, 74] and on a seasonal scale to exploit seasonal resources [90]. The overall complexity of coyote response to human modification illustrates how a species’ response to novel environments can occur on multiple scales. These responses highlight differences in behavioral plasticity capacity between the two species.

Focusing on multiple aspects of space-use by investigating home range size and habitat selection behaviors including spatial, temporal, and individual variation allowed us to reveal the complexity and differences in the responses of two carnivores to anthropogenic disturbance that moves beyond comparing fine-scale and broad-scale habitat selection. While investigating functional responses in resource selection is common [21, 26], investigating temporal variation in these functional responses is rarely done, yet considering this aspect is critical in understanding the degree of nuance in spatial behavior. However, using these characteristics allowed us to categorize species based on home range size, habitat selection, and functional response behaviors and highlight how a species is responding to anthropogenic change by combining investigations of functional responses in home range size and resource selection with selection behaviors on spatial and temporal scales.

Finding how species or populations adjust their space use behaviors to respond to features in their environment can have important conservation and management implications. As human modification continues, understanding the full extent of its effects on wildlife population dynamics and fitness [89] as a result of individual- and population-level responses is increasingly crucial. Species that are less plastic are more likely to be disadvantaged in high-disturbance environments, while behavioral flexibility leads to increased success and tolerance of anthropogenic environments [45, 46]. For example, bobcat populations in North America only recently began recovering after record lows in the 1900’s [75], while coyote populations have both increased in number and range across North America with anthropogenic land changes and extirpation of large predators [38, 43], illustrating the implications of plasticity and tolerance to human modification. However, while a nuanced response to human modification can provide benefits in exploiting anthropogenic habitat, there are also risks associated with this behavior. Forty-two percent of the coyotes tracked in this study (*n* = 13) were killed (hunted or trapped) within one year after being collared. While coyote abundance in this population appeared to remain stable despite these mortalities, there remains a risk to individuals living with humans.

## Conclusions

The variation in complexity in spatiotemporal response described here could be used as another metric to predict how species will react to future changes, and potentially as to how to best manage them. Rettie and Messier [72] proposed the “hierarchy of limiting factors” hypothesis, stating that species will display space-use response at a broader scale to address their most limiting factors. Similar to this idea, species on the broad-scale end of the spectrum appear to respond to human development by displaying broad spatial response, indicating that habitat itself might be their biggest limiting factor [72]. As such, managing species like bobcats should focus on habitat manipulation to mitigate blanket responses in home range size and habitat selection. Species on the fine-scale end of the spectrum, like coyotes, may respond more to factors impacting the type of interactions with humans, such as harvest management, because they can be more flexible in habitat use and risk avoidance on a temporal scale. In such, our results highlight the importance of investigating spatial, temporal and individual responses to elucidate how other species might be impacted by human activities and how to best mitigate these activities.

### Supplementary Information

Additional file 1.

## Data Availability

Data will be archived upon acceptance in a FigShare repository.
