# Supplementary material for: Carnivore space use behaviors reveal variation in responses to human land modification
Source: Mov Ecol. 2024 Jul 18;12:51. doi: 10.1186/s40462-024-00493-7 (PMC11256472; doi:10.1186/s40462-024-00493-7)
Supplement: Supplementary file 1 — Additional file 1. [file 40462_2024_493_MOESM1_ESM.docx]

**Supporting Information**

Appendix 1. Metadata

Table S.1. Information on demographics, GPS fixes, and land cover use for each individual bobcat and coyote included.

| **ID #** | **Species** | **Sex** | **Study Site** | **# Usable GPS Locs** | **GPS Fix Success Rate** | **% Locs in Forest** | **% Locs in Exurban** | **% Locs in Ag.** | **% Locs in Other** |
| --- | --- | --- | --- | --- | --- | --- | --- | --- | --- |
| 81460 | bobcat | female | south | 988 | 0.70 | 89.98 | 4.86 | 3.85 | 1.32 |
| 81459 | coyote | male | central | 1579 | 0.72 | 29.89 | 2.60 | 61.24 | 6.27 |
| 81458 | coyote | male | central | 1172 | 0.79 | 9.98 | 5.63 | 81.06 | 3.33 |
| 81454 | coyote | male | south | 213 | 0.57 | 86.85 | 0.00 | 11.27 | 1.88 |
| 81455 | coyote | female | south | 600 | 0.53 | 85.67 | 4.33 | 8.33 | 1.67 |
| 81449 | bobcat | female | south | 293 | 0.47 | 95.22 | 2.05 | 2.39 | 0.34 |
| 81448 | bobcat | male | south | 775 | 0.56 | 70.97 | 6.97 | 22.06 | 0.00 |
| 81447 | bobcat | female | south | 566 | 0.40 | 93.11 | 6.71 | 0.18 | 0.00 |
| 81453 | coyote | male | central | 2284 | 0.72 | 22.72 | 3.94 | 73.07 | 0.26 |
| 81445 | coyote | male | central | 561 | 0.69 | 30.48 | 5.53 | 56.86 | 7.13 |
| 81462 | coyote | female | central | 308 | 0.18 | 0.00 | 2.92 | 97.08 | 0.00 |
| 81463 | coyote | male | central | 1895 | 0.77 | 43.27 | 4.06 | 49.13 | 3.54 |
| 81457 | coyote | male | central | 1447 | 0.61 | 51.55 | 0.62 | 47.06 | 0.76 |
| 81464 | coyote | male | central | 2404 | 0.83 | 8.90 | 6.82 | 83.82 | 0.46 |
| 81456 | coyote | male | central | 1072 | 0.61 | 32.93 | 0.75 | 64.74 | 1.59 |
| 81461 | coyote | male | central | 1049 | 0.72 | 21.26 | 2.00 | 75.88 | 0.86 |
| 81446 | bobcat | female | central | 841 | 0.51 | 43.16 | 9.39 | 43.52 | 3.92 |
| 85786 | coyote | female | south | 1138 | 0.69 | 82.25 | 0.70 | 16.26 | 0.79 |
| 84141 | bobcat | female | south | 2352 | 0.73 | 90.73 | 3.70 | 3.96 | 1.62 |
| 81450 | bobcat | male | south | 1214 | 0.50 | 93.41 | 5.52 | 0.00 | 1.07 |
| 84142 | bobcat | female | south | 2695 | 0.64 | 78.66 | 2.93 | 16.33 | 2.08 |
| 84143B | coyote | male | central | 3281 | 0.79 | 29.82 | 2.87 | 64.76 | 2.56 |
| 85784 | coyote | male | central | 3596 | 0.88 | 3.64 | 1.56 | 94.10 | 0.70 |
| 85792 | coyote | male | central | 2265 | 0.72 | 32.64 | 2.12 | 56.18 | 9.05 |
| 85790 | coyote | female | central | 2511 | 0.70 | 42.19 | 4.46 | 48.88 | 4.46 |
| 85791 | coyote | female | central | 1130 | 0.82 | 34.99 | 2.30 | 57.75 | 4.96 |
| 85793 | coyote | female | central | 3092 | 0.80 | 10.68 | 1.13 | 77.74 | 10.45 |
| 85788 | coyote | female | central | 2273 | 0.69 | 28.42 | 12.19 | 55.43 | 3.96 |
| 84144 | bobcat | male | central | 2556 | 0.65 | 65.34 | 0.23 | 24.96 | 5.48 |
| 85795 | bobcat | male | central | 1799 | 0.56 | 55.39 | 12.57 | 28.75 | 3.28 |
| 85785 | coyote | male | south | 1845 | 0.63 | 88.62 | 1.79 | 6.50 | 3.09 |
| 87643 | coyote | female | south | 1527 | 0.64 | 88.79 | 3.60 | 6.49 | 1.11 |
| 85789 | coyote | female | south | 2285 | 0.68 | 67.86 | 1.18 | 27.98 | 2.98 |
| 87649 | coyote | male | south | 1570 | 0.65 | 87.00 | 1.02 | 10.39 | 1.59 |
| 87648 | coyote | female | south | 459 | 0.77 | 83.41 | 4.59 | 11.57 | 0.44 |
| 87638 | bobcat | male | south | 1539 | 0.47 | 89.99 | 4.16 | 1.95 | 3.90 |
| 87640 | bobcat | male | south | 778 | 0.66 | 63.45 | 24.32 | 10.17 | 2.06 |
| 87639 | bobcat | female | south | 474 | 0.64 | 80.13 | 3.59 | 16.07 | 0.21 |
| 87636 | bobcat | male | south | 1975 | 0.58 | 68.30 | 9.57 | 18.13 | 4.00 |
| 85787B | coyote | female | central | 1346 | 0.78 | 21.12 | 7.29 | 69.89 | 1.71 |
| 85794 | coyote | male | central | 2658 | 0.77 | 20.21 | 5.27 | 71.55 | 2.97 |
| 87647 | coyote | male | central | 1655 | 0.63 | 48.28 | 0.73 | 29.37 | 21.63 |
| 87644A | coyote | male | central | 543 | 0.83 | 50.83 | 1.10 | 40.33 | 7.73 |
| 87646 | coyote | male | central | 3175 | 0.77 | 23.56 | 1.32 | 70.77 | 4.35 |
| 87650 | coyote | male | central | 2871 | 0.74 | 25.84 | 1.18 | 71.96 | 1.01 |
| 87641 | bobcat | male | central | 2108 | 0.59 | 47.89 | 12.06 | 35.64 | 4.41 |

Appendix 2. Temporal periods

Table S.2. Diel period delineations of day, night, and crepuscular periods by minute throughout the calendar year, based on sunrise and sunset times in Illinois (Central Time Zone).

| Dates | Day | Night | Crepuscular |
| --- | --- | --- | --- |
| 12/21-3/19 | 09:04-16:56 | 19:56-06:04 | 06:04-09:04, 16:56-19:57 |
| 3/20-6/20 | 08:16-18:46 | 21:46-05:16 | 05:15-08:16, 18:46-21:46 |
| 6/21-9/21 | 08:08-18:39 | 21:39-05:08 | 05:08-08:08, 18:39-21:39 |
| 9/22-12/20 | 08:56-16:55 | 19:55-05:56 | 05:56-08:56, 16:55-19:55 |

Table S.3. Ecological season delineations for bobcats (*n*=14) and coyotes (*n*=28) in Illinois by date. Four bobcat and four coyote seasons were divided using location data from 2018-2021.

| Bobcat | Early Winter | Late Winter | Spring/Summer | Fall |
| --- | --- | --- | --- | --- |
|  | 11/6-12/3 | 12/4-1/11 | 1/12-10/13 | 10/14-11/5 |
| Coyote | Early Winter | Late Winter | Spring | Summer/Fall |
|  | 12/3-1/7 | 1/8-1/25 | 1/26-6/18 | 6/19-12/2 |

Appendix 3. Additional results and uncertainty in home range sizes

Figure S.1. Bobcat (*n*=11) and coyote (*n*=24) home range size maximum and minimum estimations using AKDE (minimum bobcat μ=26.9 km^2^, coyote μ=138.4 km^2^, t=-2.817, DF=33, p=0.008; maximum bobcat μ=38.2 km^2^, coyote μ=330.1 km^2^, t=-2.778, DF=33, p=0.009).

Figure S.2. Results of sex and site comparison t-tests for bobcats and coyotes. There was not a significant difference in home range size between bobcat males and females (panel A, female *n*=6, male *n*=5, t=-1.415, DF=9, p=0.191), but home ranges in central Illinois were significantly larger than those in southern Illinois (panel B, central Illinois *n*=2, μ=115.1 km^2^, southern Illinois *n*=9, μ=13.9 km^2^, t=3.868, DF=9, p=0.004). There was also no significant difference between male and female coyote home range size (panel C, female *n*=10, male *n*=14, t=-0.401, DF=22, p=0.692) or between study sites (panel D, central Illinois *n*=18, southern Illinois *n*=6, t=0.583, DF=22, p=0.566).

Figure S.3. Bobcat (female *n*=6, male *n*=5) AKDE home range size minimum and maximum estimations by sex (minimum t=-1.545, DF=9, p=0.157; maximum t=-1.312, DF=9, p=0.222).

Figure S.4. Bobcat (central Illinois *n*=2, southern Illinois *n*=9) AKDE home range size minimum and maximum estimations by study site (minimum central μ=96.3 km^2^, southern μ=11.5 km^2^, t=4.049, DF=9, p=0.003; maximum central μ=135.5 km^2^, southern μ=16.5 km^2^, t=3.723, DF=9, p=0.004).

Figure S.5. Coyote (female *n*=10, male *n*=14) AKDE home range size minimum and maximum estimations by sex (minimum t=-0.519, DF=22, p=0.609; maximum t=-0.327, DF=22, p=0.747).

Figure S.6. Coyote (central Illinois *n*=18, southern Illinois *n*=6) AKDE home range size minimum and maximum estimations by study site (minimum t=0.758, DF=22, p=0.457; maximum t=0.482, DF=22, p=0.635).

Appendix 4. AIC_C_ table of home range size in response to human modification candidate models

Table S.4. AIC_C_ table of three regression candidate models representing competing hypotheses regarding the relationship of home range size and the amount of human modification within the home range for bobcats and coyotes.

|  | Bobcat | |  | Coyote | |  |
| --- | --- | --- | --- | --- | --- | --- |
| Model | AIC_C_ | ΔAIC_C_ | AIC_C_ ω | AIC_C_ | ΔAIC_C_ | AIC_C_ ω |
| Intercept | 21.93 | 8.07 | 0.02 | 56.30 | 0.00 | 0.56 |
| Linear | 13.86 | 0.00 | 0.86 | 57.29 | 0.99 | 0.34 |
| Quadratic | 17.76 | 3.90 | 0.12 | 59.71 | 0.10 | 0.10 |

Appendix 5. Individual-level RSF in response to human modification complete results

Table S.5. AIC_C_ table of the top models (intercept-only, linear, or quadratic) for each bobcat and coyote covariate (agricultural, exurban, other landcover, distance to water, and distance to road) and temporal period and the means for all temporal periods for regressions of individual-level RSF coefficients in response to the proportion of human modification within each individual’s home range. Bobcat temporal periods include day, crepuscular, and night for each ecological season of spring/summer, fall, and late winter in addition to all times of day in late winter combined. Coyote temporal periods include day, crepuscular, and night for each ecological season of spring, summer/fall, early winter, and late winter. The top model is shown and the ΔAIC_C_ between the top model and next top model are shown when the top model is the linear or quadratic regression.

| Species | Covariate | Temporal Period | Top Model | ΔAIC_C_ to Second Model |
| --- | --- | --- | --- | --- |
| Bobcat | Agriculture | Mean | Intercept |  |
|  |  | Spring/Summer Day | Quadratic | 3.17 |
|  |  | Spring/Summer Crep | Linear | 6.64 |
|  |  | Spring/Summer Night | Linear | 4.07 |
|  |  | Fall Day | Intercept |  |
|  |  | Fall Crep | Intercept |  |
|  |  | Fall Night | Intercept |  |
|  |  | Late Winter Day | Intercept |  |
|  |  | Late Winter Crep | Intercept |  |
|  |  | Late Winter Night | Intercept |  |
|  | Exurban | Mean | Intercept |  |
|  |  | Spring/Summer Day | Intercept |  |
|  |  | Spring/Summer Crep | Intercept |  |
|  |  | Spring/Summer Night | Linear | 2.22 |
|  |  | Fall Day | Intercept |  |
|  |  | Fall Crep | Intercept |  |
|  |  | Fall Night | Intercept |  |
|  |  | Late Winter Day | Intercept |  |
|  |  | Late Winter Crep | Intercept |  |
|  |  | Late Winter Night | Intercept |  |
|  | Other | Mean | Linear | 3.28 |
|  |  | Spring/Summer Day | Linear | 5.85 |
|  |  | Spring/Summer Crep | Linear | 3.07 |
|  |  | Spring/Summer Night | Intercept |  |
|  |  | Fall Day | Intercept |  |
|  |  | Fall Crep | Intercept |  |
|  |  | Fall Night | Intercept |  |
|  |  | Late Winter Day | Intercept |  |
|  |  | Late Winter Crep | Intercept |  |
|  |  | Late Winter Night | Intercept |  |
|  | Dist. to Water | Mean | Linear | 3.27 |
|  |  | Spring/Summer Day | Linear | 2.27 |
|  |  | Spring/Summer Crep | Linear | 3.55 |
|  |  | Spring/Summer Night | Intercept |  |
|  |  | Fall Day | Intercept |  |
|  |  | Fall Crep | Intercept |  |
|  |  | Fall Night | Intercept |  |
|  |  | Late Winter Day | Intercept |  |
|  |  | Late Winter Crep | Intercept |  |
|  |  | Late Winter Night | Intercept |  |
|  | Dist. to Road | Mean | Intercept |  |
|  |  | Spring/Summer Day | Intercept |  |
|  |  | Spring/Summer Crep | Intercept |  |
|  |  | Spring/Summer Night | Intercept |  |
|  |  | Fall Day | Intercept |  |
|  |  | Fall Crep | Intercept |  |
|  |  | Fall Night | Intercept |  |
|  |  | Late Winter Day | Intercept |  |
|  |  | Late Winter Crep | Intercept |  |
|  |  | Late Winter Night | Intercept |  |
| Coyote | Agriculture | Mean | Intercept |  |
|  |  | Spring Day | Linear | 2.02 |
|  |  | Spring Crep | Linear | 2.19 |
|  |  | Spring Night | Linear | 2.66 |
|  |  | Summer/Fall Day | Intercept |  |
|  |  | Summer/Fall Crep | Intercept |  |
|  |  | Summer/Fall Night | Linear | 2.18 |
|  |  | Early Winter Day | Linear | 2.09 |
|  |  | Early Winter Crep | Intercept |  |
|  |  | Early Winter Night | Intercept |  |
|  |  | Late Winter Day | Intercept |  |
|  |  | Late Winter Crep | Intercept |  |
|  |  | Late Winter Night | Intercept |  |
|  | Exurban | Mean | Linear | 6.56 |
|  |  | Spring Day | Linear | 10.28 |
|  |  | Spring Crep | Intercept |  |
|  |  | Spring Night | Intercept |  |
|  |  | Summer/Fall Day | Intercept |  |
|  |  | Summer/Fall Crep | Intercept |  |
|  |  | Summer/Fall Night | Intercept |  |
|  |  | Early Winter Day | Intercept |  |
|  |  | Early Winter Crep | Intercept |  |
|  |  | Early Winter Night | Intercept |  |
|  |  | Late Winter Day | Intercept |  |
|  |  | Late Winter Crep | Quadratic | 4.62 |
|  |  | Late Winter Night | Intercept |  |
|  | Other | Mean | Quadratic | 11.75 |
|  |  | Spring Day | Intercept |  |
|  |  | Spring Crep | Intercept |  |
|  |  | Spring Night | Intercept |  |
|  |  | Summer/Fall Day | Intercept |  |
|  |  | Summer/Fall Crep | Quadratic | 2.51 |
|  |  | Summer/Fall Night | Quadratic | 6.02 |
|  |  | Early Winter Day | Linear | 3.15 |
|  |  | Early Winter Crep | Intercept |  |
|  |  | Early Winter Night | Intercept |  |
|  |  | Late Winter Day | Linear | 4.96 |
|  |  | Late Winter Crep | Linear | 5.17 |
|  |  | Late Winter Night | Intercept |  |
|  | Dist. to Water | Mean | Quadratic | 4.87 |
|  |  | Spring Day | Linear | 10.63 |
|  |  | Spring Crep | Linear | 4.69 |
|  |  | Spring Night | Linear | 2.38 |
|  |  | Summer/Fall Day | Intercept |  |
|  |  | Summer/Fall Crep | Quadratic | 6.73 |
|  |  | Summer/Fall Night | Intercept |  |
|  |  | Early Winter Day | Intercept |  |
|  |  | Early Winter Crep | Intercept |  |
|  |  | Early Winter Night | Intercept |  |
|  |  | Late Winter Day | Intercept |  |
|  |  | Late Winter Crep | Intercept |  |
|  |  | Late Winter Night | Intercept |  |
|  | Dist. to Road | Mean | Quadratic | 2.10 |
|  |  | Spring Day | Intercept |  |
|  |  | Spring Crep | Intercept |  |
|  |  | Spring Night | Intercept |  |
|  |  | Summer/Fall Day | Intercept |  |
|  |  | Summer/Fall Crep | Intercept |  |
|  |  | Summer/Fall Night | Intercept |  |
|  |  | Early Winter Day | Intercept |  |
|  |  | Early Winter Crep | Intercept |  |
|  |  | Early Winter Night | Intercept |  |
|  |  | Late Winter Day | Intercept |  |
|  |  | Late Winter Crep | Intercept |  |
|  |  | Late Winter Night | Intercept |  |


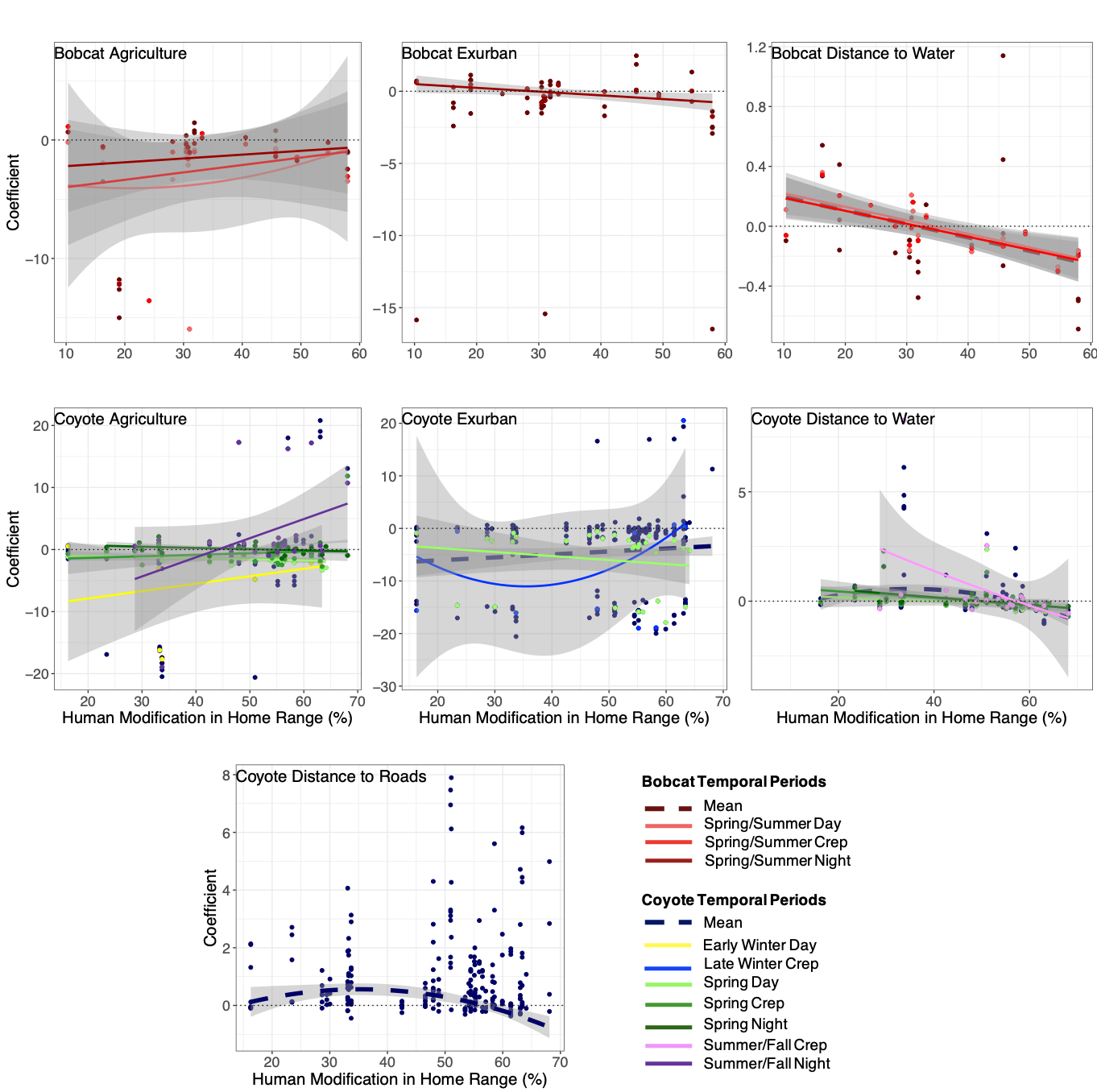


Figure S.7. Top linear and quadratic regression model trendlines for bobcat and coyote individual-level RSF coefficients for agricultural landcover, exurban landcover, and distance to water in reference to forested landcover in response to the proportion of human modification present in each individual’s home range. Each line is a trend for a specific temporal period (solid) or a mean for all temporal periods (bolded and dashed). Confidence interval shadows shown for each trendline. Individual coefficient points are shown in the corresponding temporal period color if their trendline is shown. Points not corresponding to a top temporal period model are shown in the same color as the mean trendline.
